# Treatment decisions in estrogen receptor-positive early breast cancer patients with intermediate onco*type* DX recurrence score results

**DOI:** 10.1186/2193-1801-3-71

**Published:** 2014-02-06

**Authors:** Georgeta Fried, Mor Moskovitz

**Affiliations:** Rambam Health Care Campus, 6 Ha’Aliya Street, Haifa, 31096 Israel

**Keywords:** Adjuvant chemotherapy, Breast cancer, Decision making, Intermediate risk, Onco*type* DX, Recurrence score

## Abstract

This retrospective study evaluated the impact of intermediate Recurrence Score^®^ results on adjuvant treatment decisions in estrogen receptor-positive (ER+) early invasive breast cancer, comparing treatment recommendations pre-testing with actual treatments received post-testing. Of the 111 patients included in the analysis, 78 (70.3%) had hormonal therapy (HT) and 33 (29.7%) had chemohormonal therapy (CHT) recommendations pre-testing. The Recurrence Score was significantly higher in those with a pre-testing CHT recommendation compared with those with a pre-testing HT recommendation (median of 24 vs. 22; *P* = 0.047; Mann–Whitney–Wilcoxon [MWW] test). Post-testing, treatment of 24 patients (21.6%) was different from their pre-testing recommendation. The difference between CHT recommendation rate pre-testing and the rate of CHT received post-testing was nonsignificant for the entire cohort and for patients’ subgroups (by age, tumor size, and grade) (*P* >0.17; McNemar’s test). Following classification of the cohort into two Recurrence Score subcategories (low-intermediate, [18-25]; high-intermediate, [26-30]), changes in treatment decisions (pre-testing recommendations vs. actual treatments received post testing) were reported for 16.5% of low-intermediate and 34.4% of high-intermediate patients. Post-testing, the rate of CHT decreased (by 58%) in the low-intermediate subcategory and increased (by 64%) in the high-intermediate subcategory (*P* <0.01, both subcategories). In logistic regression analyses, the Recurrence Score subcategory was the only significant predictor of changes in treatment decisions (pre-testing recommendations vs. actual treatments received post testing; *P* <0.01). The only significant difference between the two subsets of patients with such a change (HT to CHT, 11 patients; CHT to HT, 13 patients) was the Recurrence Score (median of 28 vs. 20, respectively; *P* = 0.0014; MWW test). These findings demonstrate that intermediate Recurrence Score results provide clinically relevant information and impact treatment decisions in ER + early breast cancer.

## Introduction

The Onco*type* DX^®^ Breast Cancer assay (Genomic Health, Inc., Redwood City, CA) has been validated as a prognosticator and a predictor of the likelihood of chemotherapy benefit in estrogen receptor-positive (ER+) early invasive breast cancer (Paik et al. 2004; Paik et al. 2006; Habel et al. 2006; Goldstein et al. 2008; Albain et al. 2010; Dowsett et al. 2010; Toi et al. 2010; Mamounas et al. 2012). The Onco*type* DX Recurrence Score^®^ is a continuous value ranging from 0 to 100; it can be used to classify patients into 3 risk categories: low (<18), intermediate (18-30), and high (≥31) (Paik et al. 2004). For patients with high Recurrence Score results, the benefit from adjuvant chemotherapy is significant while for patients with low Recurrence Score results, there is minimal, if any, benefit from chemotherapy (Paik et al. 2006; Albain et al. 2010). In the intermediate Recurrence Score category, adjuvant chemotherapy may confer a modest benefit for some patients (Paik et al. 2006; Albain et al. 2010). Since becoming available in 2004, numerous studies conducted around the world (United States, European countries, Japan, Australia, and Israel) have investigated its impact on treatment decision-making in clinical practice and have consistently shown that testing results in a change in treatment recommendations in 19-51% of cases (from chemohormonal therapy to hormonal therapy alone and vice versa) correlating with a net reduction in chemotherapy use (Oratz et al. 2007; Asad et al. 2008; Rayhanabad et al. 2008; Henry et al. 2009; Klang et al. 2010; Lo et al. 2010; Ademuyiwa et al. 2011; de Boer et al. 2011; Geffen et al. 2011; Kamal et al. 2011; Oratz et al. 2011; Yamauchi et al. 2011; Albanell et al. 2012; Eiermann et al. 2012; Gligorov et al. 2012; Albanell et al. 2013; Holt et al. 2013). In the low Recurrence Score category (the largest category including 40-58% of patients in these studies), the recommendation change was mostly from chemohormonal therapy to hormonal therapy alone and in the high Recurrence Score category (the smallest category including 5-21% of patients in these studies), the recommendation change was mostly from hormonal therapy to chemohormonal therapy (Oratz et al. 2007; Klang et al. 2010; Lo et al. 2010; Ademuyiwa et al. 2011; Geffen et al. 2011; Oratz et al. 2011; Albanell et al. 2012; Eiermann et al. 2012; Gligorov et al. 2012; Albanell et al. 2013; Holt et al. 2013). In the intermediate Recurrence Score category, the observed changes in treatment recommendations were not consistent across studies, and data on the impact of the Recurrence Score and clinicopathologic characteristics on treatment recommendations in this subpopulation are limited (Oratz et al. 2007; Klang et al. 2010; Lo et al. 2010; Ademuyiwa et al. 2011; Geffen et al. 2011; Oratz et al. 2011; Albanell et al. 2012; Eiermann et al. 2012; Gligorov et al. 2012; Albanell et al. 2013; Holt et al. 2013). The current study was designed to evaluate the associations between Recurrence Score results and clinicopathologic characteristics in a cohort of patients with ER + early invasive breast cancer and an intermediate Recurrence Score result and to evaluate the impact of the Recurrence Score results and clinicopathologic characteristics on treatment decisions.

## Materials and methods

### Study design and patients

This retrospective single-institution cohort study involving patients with ER + early invasive breast cancer and an intermediate Recurrence Score result included all female patients who were diagnosed and treated in the Rambam Health Care Campus (Haifa, Israel) between October 2005 and September 2010. The study was approved by the institutional review board of Rambam Health Care Campus.

### Data source

Clinicopathological characteristics, Recurrence Score results, and treatments received were obtained from patients’ records. Physicians’ treatment recommendations prior to Onco*type* DX testing were documented on requisition of the assay.

### Statistical analyses

Descriptive statistics were used to summarize clinicopathologic characteristics and Recurrence Score results. *T*-test/Mann–Whitney–Wilcoxon (MWW) and Chi-squared/Fisher’s exact tests were used, as appropriate, to compare continuous and categorical parameters, respectively, between patients treated with hormonal therapy alone and those treated with chemohormonal therapy. McNemar's test was used to assess whether the difference between the proportions of patients with hormonal and chemohormonal therapy recommendations (pre-testing) and the proportion of patients who received hormonal and chemohormonal therapy (post-testing) was significant. Logistic regression analyses were used to assess the probability of a treatment change from pre-testing hormonal therapy recommendation to post-testing treatment with chemohormonal therapy and from pre-testing hormonal therapy recommendation to post-testing treatment with chemohormonal therapy as a function of patient’s age, tumor size, tumor grade, and the Recurrence Score result. All analyses were conducted using SAS statistical software version 9. 2 (SAS Institute Inc., Cary, NC); *P* <0.05 was considered significant.

## Results

### Patient and tumor characteristics

A total of 116 patients with intermediate Recurrence Score results were included in the analysis (Table 1). Patients were followed for a median (range) of 30 (4-70) months. The majority of patients (86.1%) were node negative. Grade distribution was similar to that observed in the general patient population undergoing Onco*type* DX testing in Israel (Ben-Baruch et al. 2013). Patients’ Recurrence Score results spanned the entire intermediate Recurrence Score category (18-30) and the median Recurrence Score result was 22 (Table 1).

**Table 1:** **Baseline patient and tumor characteristics**

	All patients
	(***N*** = 116)
**Age**	
Mean (SD), years	56.2 (9.0)
Median (range), years	56.5 (35-76)
**Tumor size** ^**a**^	
Mean (SD), mm	16.4 (6.9)
Median (range), mm	15.0 (6-55)
**Nodal status,** ^**b**^ ***n*** **(%)**	
Node negative	93 (86.1)
Node positive	15 (13.9)
**Tumor grade,** ^**c**^ ***n*** **(%)**	
1	16 (15.4)
2	66 (63.5)
3	22 (21.2)
**Type of surgery,** ^**d**^ ***n*** **(%)**	
Lumpectomy	96 (83.5)
Mastectomy	19 (16.5)
**Recurrence Score**	
Mean (SD)	22.9 (3.7)
Median (range)	22 (18-30)

*SD* standard deviation.

^a^Tumor size information was unavailable for 1 patient.

^b^Nodal status information was unavailable for 8 patients.

^c^Tumor grade information was unavailable for 12 patients.

^d^Surgery type information was unavailable for 1 patient.

### Clinicopathologic characteristics and Recurrence Score results by pre-testing treatment recommendations

The analysis included 111 patients for whom information about treatment recommendations before Onco*type* DX testing and actual treatments received was available. Before testing, 78 patients (70.3%) were recommended hormonal therapy alone and 33 patients (29.7%) were recommended chemohormonal therapy. These two groups were not statistically significantly different with respect to age (*P* = 0.056; *t*-test), and had statistically significant differences with respect to tumor size (*P* = 0.0037, *t*-test), nodal involvement (*P* = 0.00013, Fisher’s exact test), grade distribution (*P* = 0.043, Chi-squared test), and surgery type (lumpectomy vs. mastectomy, *P* = 0.0015, Chi-squared test). In comparison to patients with a pre-testing recommendation of hormonal therapy alone, those with a pre-testing recommendation of chemohormonal therapy had larger tumors, and there were higher proportions of node-positive patients, grade 3 tumors, and patients who had undergone mastectomy (Table 2).

**Table 2:** **Baseline patient and tumor characteristics for patients with pre-testing recommendations of hormonal therapy alone vs. chemohormonal therapy**

	Physician’s treatment recommendation pre-Onco***type*** DX testing	
	Hormonal therapy (***n*** = 78)	Chemohormonal therapy (***n*** = 33)
**Age** ^**a**^		
Mean (SD), years	57.4 (9.0)	53.8 (9.1)
Median (range), years	57.5 (37-76)	55 (35-72)
**Tumor size** ^**b**^		
Mean (SD), mm	14.8 (5.4)	20.1 (9.1)
Median (range), mm	14 (6-35)	19 (8.5-55)
**Nodal status,** ^**c**^ ***n*** **(%)**		
Node negative	71 (94.7)	19 (63.3)
Node positive	4 (5.3)	11 (36.7)
**Tumor grade,** ^**d**^ ***n*** **(%)**		
1	14 (20.6)	1 (3.2)
2	42 (61.8)	20 (64.5)
3	12 (17.7)	10 (32.3)
**Type of surgery,** ^**e**^ ***n*** **(%)**		
Lumpectomy	71 (91.0)	22 (66.7)
Mastectomy	7 (9.0)	11 (33.3)
**Recurrence score result** ^**f**^		
Mean (SD)	22.5 (3.5)	24.1 (4.0)
Median (range)	22 (18-29)	24 (18-30)

*SD* standard deviation.

^a^ *P* = 0.056 (*t*-test for comparing means).

^b^ *P* = 0.0037 (*t*-test for comparing means). Tumor size information was unavailable for 1 patient in the chemohormonal therapy group.

^c^ *P* = 0.00013 (Fisher’s exact test). Nodal status information was unavailable for 3 patients in the hormonal therapy group and 3 patients in the chemohormonal therapy group.

^d^ *P* = 0.043 (Chi-squared test). Grade information was unavailable for 10 patients in the hormonal therapy group and 2 patients in the chemohormonal therapy group.

^e^ *P* = 0.0015 (Chi-squared test).

^f^ *P* = 0.047 (MWW test).

Notably, the group with a pre-testing chemohormonal therapy recommendation had a statistically significantly higher median Recurrence Score result than the group with a pre-testing hormonal therapy recommendation (24 vs. 22; *P* = 0.047; MWW test) (Table 2).

### Recurrence Score results and treatments received

**Figure 1:**
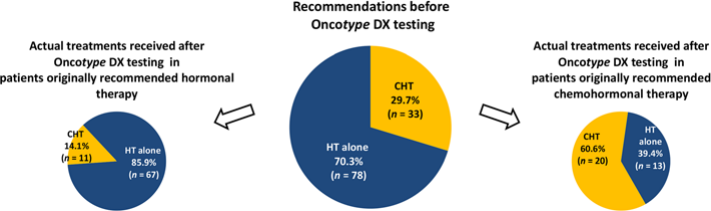
**Overall impact of knowing the Recurrence Score result on treatments received (** ***n*** **= 111).** *CHT* chemohormonal therapy; *HT* hormonal therapy.

After knowing the Recurrence Score results, 24 patients (21.6%; 95% confidence interval [CI], 13.8-29.4%) received treatment that was different from their pre-testing treatment recommendation. Of the 78 patients who were originally recommended hormonal therapy alone, 11 received chemohormonal therapy (14.1%; 95% CI, 6.2-22.0%); of the 33 patients who were originally recommended chemohormonal therapy, 13 received hormonal therapy alone (39.4%; 95% CI, 21.8-57.0%) (Figure 1). Overall, there was virtually no difference between the proportion of patients who were recommended chemohormonal therapy pre-testing and the proportion of patients who received chemohormonal therapy post-testing (29.7% before vs. 27.9% after; *P* = 0.68; McNemar's test).

Treatment changes were also analyzed by age (≤50, >50 years), tumor size (≤15 mm, >15 mm), and grade (for patients with grade 2 and 3 tumors). In all the analyzed subgroups, the net change in chemotherapy use (i.e., the difference between the proportion of patients with pre-testing chemohormonal therapy recommendations and the proportion of patients who received chemohormonal therapy post-testing) was nonsignificant (*P* values between 0.18 and 0.82; McNemar's test). The overall change rate in treatment decisions (from pre-testing chemohormonal therapy recommendation to post-testing hormonal therapy treatment and from pre-testing hormonal therapy recommendation to post-testing chemohormonal therapy treatment) was similar in the younger (≤50 years) and older (>50 years) age subgroups (16.7% [95% CI, 2.5-30.8%] vs. 23.5% [95% CI, 14.0-32.9%]), and in patients with smaller (≤15 mm) and larger (>15 mm) tumors (16.9% [95% CI, 7.6-26.3%] vs. 26.7% [95% CI, 13.2-40.1%]). Of the 16 patients with grade 1 tumors, only 1 (6.3% [95% CI, 0-30.3%]) had a treatment decision change (from pre-testing hormonal therapy recommendation to receiving chemohormonal therapy post-testing); in patients with grade 2 and grade 3 tumors, the overall change rate was more pronounced and similar (24.2% [95% CI, 13.2-35.2%] and 27.3% [95% CI, 7.1-47.5%], respectively).

**Figure 2:**
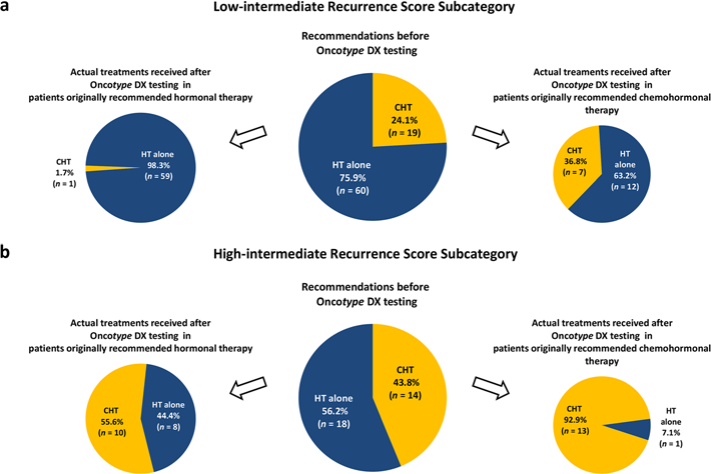
**Impact of knowing the Recurrence Score result on treatments received in the low-intermediate Recurrence Score subcategory (Recurrence Score values of 18-25;** ***n*** **= 79) (a) and in the high-intermediate Recurrence Score subcategory (Recurrence Score values of 26-30;** ***n*** **= 32) (b)** *CHT* chemohormonal therapy; *HT* hormonal therapy.

To explore whether clinicians perceive intermediate Recurrence Score patients in a uniform manner, the cohort was divided into two intermediate Recurrence Score subcategories (the low-intermediate subcategory [Recurrence Score results: 18-25] and the high-intermediate subcategory [Recurrence Score results: 26-30]; 25 was used as the cutoff to define the subcategories as it is the cutoff used in the ongoing Onco*type* DX phase 3 TAILORx and RxPONDER trials (ClinicalTrials.gov website 2013a; ClinicalTrials.gov website 2013b)), and changes between pre-testing treatment recommendations and post-testing actual treatments received were analyzed for each subcategory separately (Figure 2). Of the 79 patients with low-intermediate Recurrence Score results, 13 (16.5% [95% CI, 8.3-24.7%]) had a change while 11 (34.4% [95% CI, 17.9-50.9%]) of the 32 patients with high-intermediate Recurrence Score results had a change. Notably, in the low-intermediate-Recurrence Score subcategory, the changes were primarily from pre-testing chemohormonal therapy recommendation to post-testing treatment with hormonal therapy alone, and consequently, the overall rate of chemohormonal therapy decreased significantly after knowing the Recurrence Score results (by 58%, from 24.1% to 10.1%; *P* = 0.002; McNemar’s test). In the high-intermediate-Recurrence Score subcategory, the changes were primarily in the opposite direction (from hormonal to chemohormonal therapy), and consequently, the overall rate of chemohormonal therapy increased significantly after knowing the Recurrence Score results (by 64%, from 43.8% to 71.9%; *P* = 0.007; McNemar’s test).

To assess the impact of the clinicopathologic parameters and the Recurrence Score results on treatment changes, we modeled the probability of a change between treatment recommendation and actual treatment received as a function of age (≤50, >50 years), tumor size (≤15 mm, >15 mm), grade (1, 2, and 3), and the Recurrence Score subcategory (low-intermediate and high-intermediate, as defined above) for patients with pre-testing hormonal therapy recommendations and for patients with pre-testing chemohormonal therapy recommendations. Possible interaction between the effect of the Recurrence Score subcategory and patients’ age, tumor size, or tumor grade (each separately) on the probability of a treatment change was examined by the Breslow Day test. As the effect of the Recurrence Score subcategory was consistent in all subgroups (with the exception of one small subgroup [n = 14]), and the Breslow Day tests were nonsignificant (*P* >0.25), only main effects were considered for the logistic regression analyses. For patients with a pre-testing hormonal therapy recommendation, the only significant predictor of a treatment change was high-intermediate Recurrence Score subcategory (odds ratio [OR], 73.8 [95% CI, 8.3-655.2] vs. the low-intermediate subcategory; *P* = 0.0001). For patients with a pre-testing chemohormonal therapy recommendation, multivariate logistic regression analysis was not technically possible because there were only 33 such patients. For these patients, univariate analysis was performed and demonstrated that the low-intermediate Recurrence Score subcategory was a significant predictor of a treatment change (OR, 22.3 [95% CI, 2.4-208.8] vs. the high-intermediate subcategory; *P* = 0.007).

Evaluation of the two subsets of patients with a treatment change (11 patients with a pre-testing hormonal therapy recommendation who were treated with chemohormonal therapy post-testing, and 13 patients with a pre-testing chemohormonal therapy recommendation who were treated with hormonal therapy post-testing) revealed no statistically significant differences in clinicopathologic parameters including patients’ age, tumor size, nodal status, tumor grade, and type of surgery performed between the groups (although the small sample size limited our ability to draw conclusions) (Table 3). Statistically significant differences between the groups were noted with respect to Recurrence Score results with higher Recurrence Score results in patients with hormonal to chemohormonal therapy change than in patients with the reverse change (median [range] of 28 [23-29] and 20 [18-28], respectively; *P* = 0.0014; MWW test) (Table 3).

**Table 3:** **Clinicopathologic and Recurrence Score results in patients with a treatment change (** ***n*** **= 24)**

	Type of treatment change	
	Pre-testing: HT. Post-testing: CHT.	Pre-testing: CHT. Post-testing: HT.
	(***n*** = 11)	(***n*** = 13)
**Age** ^**a**^		
Mean (SD), years	54.2 (3.4)	55.1 (9.5)
Median (range), years	54 (46-58)	56 (35-72)
**Tumor size** ^**b**^		
Mean (SD), mm	15.6 (5.7)	20.4 (7.6)
Median (range), mm	14 (10-28)	20.5 (10-32)
**Nodal status,** ^**c**^ ***n*** **(%)**		
Node negative	10 (100)	8 (72.7)
Node positive	0 (0)	3 (27.3)
**Tumor grade,** ^**d**^ ***n*** **(%)**		
1	1 (9.1)	0 (0)
2	8 (72.7)	7 (63.6)
3	2 (18.2)	4 (36.4)
**Type of surgery,** ^**e**^ ***n*** **(%)**		
Lumpectomy	10 (90.9)	9 (69.2)
Mastectomy	1 (9.1)	4 (30.8)
**Recurrence Score result** ^**f**^		
Mean (SD)	27.3 (1.7)	21.1 (3.0)
Median (range)	28 (23-29)	20 (18-28)

*CHT* chemohormonal therapy; *HT* hormonal therapy; *SD* standard deviation.

^a^ *P* = 0.76 (*t*-test for comparing means).

^b^ *P* = 0.11 (*t*-test for comparing means). Tumor size information was unavailable for 1 patient in the chemohormonal to hormonal therapy group.

^c^ *P* = 0.21 (Fisher’s exact test). Nodal status information was unavailable for 1 patient in the hormonal therapy to chemohormonal therapy group and 2 patients in the chemohormonal to hormonal therapy group.

^d^ *P* = 0.64 (Fisher’s exact test). Grade information was unavailable for 2 patients in the chemohormonal to hormonal therapy group.

^e^ *P* = 0.33 (Fisher’s exact test).

^f^ *P* = 0.0014 (MWW test).

## Discussion

Onco*type* DX testing is routinely used in clinical practice in Israel to support clinical decision making for patients with ER + early invasive breast cancer. The current analysis focused on the impact of intermediate Recurrence Score results on adjuvant treatment decisions in ER + early invasive breast cancer in real-life clinical practice and demonstrated that intermediate results led to a change between pre-testing recommendations and actual treatments received in approximately one-fifth (21.6%) of the cohort. The observed changes were in both directions (from hormonal therapy alone to chemohormonal therapy and vice versa) with no significant net change in chemotherapy use (30% recommended pre-testing vs. 28% actually used). Unlike low- and high-Recurrence Score patients, for whom the impact of the Recurrence Score results on treatment decisions has been shown to be consistent across studies (increased and decreased chemotherapy use, respectively) (Oratz et al. 2007; Klang et al. 2010; Lo et al. 2010; Ademuyiwa et al. 2011; Geffen et al. 2011; Oratz et al. 2011; Albanell et al. 2012; Eiermann et al. 2012; Gligorov et al. 2012; Albanell et al. 2013; Holt et al. 2013), in intermediate-Recurrence Score patients, the overall impact of the Recurrence Score results on chemotherapy use is not consistent across studies and the overall net change ranged from an increased use of chemotherapy by more than 85% to a decreased use of chemotherapy by more than 40% (Oratz et al. 2007; Klang et al. 2010; Lo et al. 2010; Ademuyiwa et al. 2011; Geffen et al. 2011; Oratz et al. 2011; Albanell et al. 2012; Eiermann et al. 2012; Gligorov et al. 2012; Albanell et al. 2013; Holt et al. 2013). These results are consistent with the hypothesis that clinicians weigh additional factors (e.g., the exact Recurrence Score value and not the Recurrence Score category, age, grade, nodal status, etc.) in considering chemotherapy for intermediate-Recurrence Score patients.

Our study evaluated the factors that may influence changes in treatment decisions in intermediate-Recurrence Score patients. Traditional parameters such as patient’s age, tumor size, and tumor grade, did not have a statistically significant impact on the change in chemotherapy use from pre- to post-Onco*type* DX testing. Notably, the Recurrence Score itself did have a significant impact. In patients who had a treatment change (pre-testing recommendation vs. actual treatment received), higher intermediate Recurrence Score results were significantly associated with a decision to treat with chemohormonal therapy, suggesting that clinicians evaluate the Recurrence Score as a continuous parameter and do not consider all intermediate-Recurrence Score patients as having the same risk of recurrence. Also, in an analysis of treatment change by Recurrence Score subcategory (using a cutoff of 25, which is the cutoff used in the ongoing Onco*type* DX phase 3 TAILORx and RxPONDER trials that were designed to assess chemotherapy benefit in node-negative patients with Recurrence Score results between 12 and 25 and node-positive patients with results ≤25, respectively (ClinicalTrials.gov website 2013a; ClinicalTrials.gov website 2013b)), the treatment changes were statistically significant with a decrease in chemotherapy use in the low-intermediate subcategory (58% relative decrease) and an increase in chemotherapy use in the high-intermediate subcategory (64% relative increase). This novel finding was also supported by a logistic regression analysis demonstrating the statistical significance of the Recurrence Score subcategory as a predictor of a treatment change.

Onco*type* DX testing has been shown to increase clinicians’ confidence in their treatment decisions (Lo et al. 2010; Albanell et al. 2012; Eiermann et al. 2012); only one study analyzed this increase by patients’ Recurrence Score results, and demonstrated that a higher proportion of clinicians reported increased confidence when the patients had low or high Recurrence Score results than when the patients had intermediate Recurrence Score results (Eiermann et al. 2012). In addition, several studies have recently shown that patients experienced significantly lower conflict about their treatment decisions and decreased situational anxiety after receiving their Recurrence Score results (Lo et al. 2010; In et al. 2011; Eiermann et al. 2012). Only one study evaluated the impact of intermediate Recurrence Score results on patients and found that among patients who preferred a passive role in their care (but not among those who preferred an active/shared role), those with intermediate Recurrence Score results had higher cancer-related distress than patients with low/high Recurrence Score (Sulayman et al. 2012), possibly reflecting the uncertainty associated with the potential benefit of chemotherapy in these patients. Thus, it may be of interest to assess the impact of intermediate Recurrence Score results (as a continuous parameter) on clinicians’ confidence as well as on patients’ psychosocial status.

The limitations of this study include its retrospective design and a relatively small sample size. In addition, since this was a single-center study, its ability to represent treatment patterns in the general breast cancer patient population in Israel may be limited.

In summary, our findings demonstrate that intermediate Recurrence Score results are not perceived in a uniform manner by clinicians, but rather provide clinically relevant information that impacts adjuvant treatment decision-making in patients with ER + early invasive breast cancer.
